# Supplementary figures and images for: Genome-Scale Metabolic Model of the Human Pathogen Candida albicans: A Promising Platform for Drug Target Prediction
Source: J Fungi (Basel). 2020 Sep 11;6(3):171. doi: 10.3390/jof6030171 (PMC7559133; doi:10.3390/jof6030171)

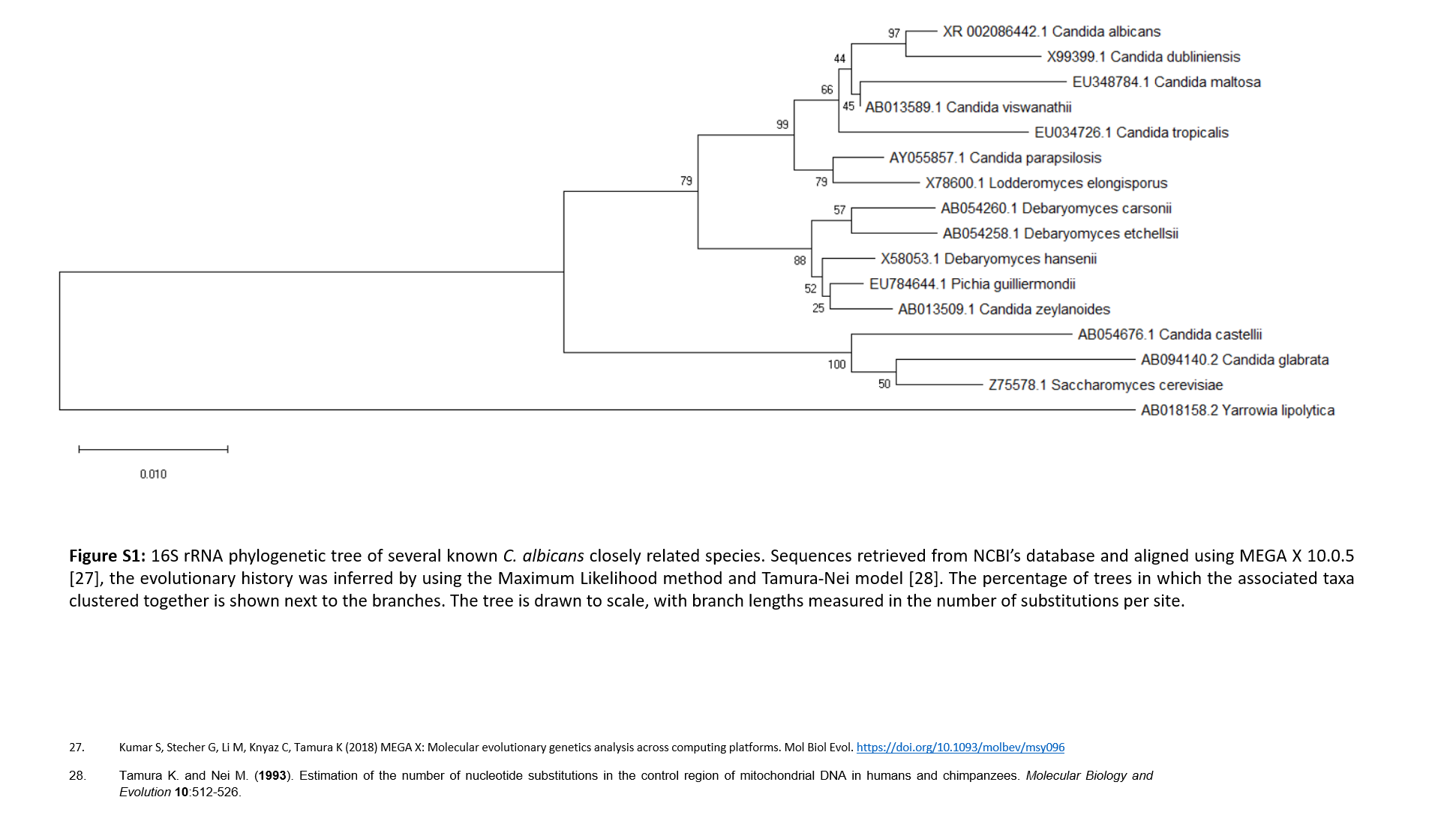

Supplement: Supplementary file 1 [file jof-06-00171-s001.zip › Figure S1 - C. albicans phylogenetic tree.png]
